# *Corynebacterium* Infection Following Implant-based Breast Reconstruction: Lessons from the Five Consecutive Patients

**DOI:** 10.1055/a-2734-7103

**Published:** 2026-01-30

**Authors:** Jiye Kim, Minwoo Park, Seung Yong Song

**Affiliations:** 1 Department of Plastic and Reconstructive Surgery, Wonju Severance Christian Hospital, Yonsei University Wonju College of Medicine, Wonju, Korea; 2 Department of Plastic and Reconstructive Surgery, Yonsei University College of Medicine, Seoul, Korea

**Keywords:** breast implants, breast reconstruction, infections, *Corynebacterium*

## Abstract

**Background:**

Infection is a challenging complication in prosthetic breast reconstruction. Recently, we encountered cases of *Corynebacterium striatum* infection following prosthetic breast reconstruction in breast cancer patients. We would like to share our experience of preventing reconstruction failure from this uncommon infection.

**Methods:**

This is a retrospective study on consecutive cases of *Corynebacterium* infection that occurred within 2 months in five patients who underwent implant-based breast reconstruction. One patient had a history of preoperative radiation therapy, one required reoperation due to postoperative bleeding, and two experienced hemovac obstruction events. Patients were immediately hospitalized and treated with hemovac insertion, IV antibiotics, and, if necessary, reoperation for salvage.

**Results:**

*Corynebacterium striatum* was identified in the hemovac cultures or intraoperative specimens of all patients. Based on the antibiotic susceptibility of the identified strain, infection with a single bacterial strain was suspected. All patients underwent hemovac re-insertion, and one patient was successfully salvaged with IV antibiotic treatment alone. However, the remaining four patients required implant change during the salvage process. The bacteria became negative within a period ranging from 1 to 7 weeks after identification, and salvage was successfully achieved without reconstruction failure in all patients by postoperative days 20 to 58.

**Conclusion:**

*Corynebacterium* is an uncommon pathogen in implant-based breast reconstruction, and reports of its treatment are very rare. This study presents our experience with successful treatment and outlines the clinical symptoms and treatment methods. We hope that this report can serve as a reference for reconstructive surgeons who may encounter similar situations.

## Introduction

Infection in implant-based breast reconstruction is a serious complication, with occurrence rates estimated between 5 and 14%, though some studies report rates as high as 30%. 1 2 This rate is not negligible and warrants attention, especially as infections requiring explantation are among the most severe complications associated with this reconstruction method.

Typically, infections associated with breast implants are predominantly caused by *Staphylococcus* species. In this case, a combination of antimicrobial therapy and the management of concurrent complications play a crucial role in infection management. It is essential to first assess for signs of infection, and if infection is suspected, obtain culture samples to determine antibiotic susceptibility. While awaiting culture results, empiric antibiotics considering broad-spectrum bacterial coverage should be initiated promptly. In cases of mild infection, oral first-generation cephalosporins or clindamycin can be considered. For moderate to severe infections, intravenous cefazolin can be administered, with intravenous vancomycin used if there is a risk of MRSA infection.

Antibiotic therapy is typically continued for 10 to 14 days, with adjustments made according to culture and susceptibility results. If there is no clinical improvement despite intravenous antibiotic therapy, surgical debridement and implant removal should be considered. Patients who undergo implant removal and debridement should receive a 10- to 14-day course of antibiotics, tailored to intraoperative culture results. Delayed reimplantation is recommended at least 4 to 6 months after completing antibiotic therapy to minimize the risk of reinfection. 2 3 4

*Corynebacterium* is a genus of gram-positive, aerobic, non-sporulating rod-shaped bacteria commonly found in nature and as part of the normal human skin and mucous membrane flora. While *Corynebacterium diphtheriae* is well known for causing diphtheria, *Corynebacterium striatum* lacks diphtheria toxin production but has emerged as an opportunistic pathogen. *C. striatum* is capable of causing a range of serious infections, including endocarditis, 5 6 prosthetic joint infections, 7 and urinary tract infections. 8 Effective treatment is often challenging due to its intrinsic resistance to multiple antibiotics. 9 10

Recently, the authors encountered an infection caused by *Corynebacterium striatum* in breast cancer patients following implant-based breast reconstruction within 2 months. A literature review reveals a lack of information on the treatment and outcomes of such infections. Therefore, we report the clinical presentation, attempted treatments, and outcomes of these cases, hoping that this report may serve as a reference for similar situations in the future.

## Methods

From October to December 2023, a retrospective review was conducted at a single center on five patients who had undergone mastectomy for breast cancer followed by implant-based reconstruction and subsequently developed clinically significant infections. *Corynebacterium striatum* was identified in either the surgical site culture or hemovac drainage culture of each patient. The patient numbers were assigned in order of the dates on which the reconstruction surgeries were performed, starting with the earliest. The Institutional Review Board of Severance Hospital (IRB No. 4–2025–0061) approved the protocol of this study. The patients provided written informed consent for the publication and the use of their images.

At our institution, the protocol for intravenous antibiotic use in patients undergoing implant-based breast reconstruction is as follows. Prophylactic antibiotics with 1 g of cefazolin are administered within 30 to 60 minutes prior to incision. Postoperatively, cefazolin 1 g is given twice daily for up to 24 hours. No antibiotics are administered beyond 24 hours after surgery. The routine protocol for our implant-based breast reconstruction surgery is as follows. All procedures were performed under sterile conditions. After betadine preparation, the surgical field was draped, and the nipple–areolar complex was sealed with an Ioban (3M, St. Paul, MN, USA) drape before making an incision or opening a sealed incision site. A range of sizer implants was inserted into the prepectoral plane to determine the optimal size. The surgical field was then irrigated with a solution containing gentamicin and cefazolin, followed by an additional betadine preparation around the skin incision. Mentor smooth round cohesive gel mammary implants (Mentor Worldwide LLC, Santa Barbara, CA, USA) were fully wrapped in an acellular dermal matrix (ADM) without any slits before being placed in the prepectoral plane. The used ADMs were MegaDerm (L&C Bio, Seongnam-Si, Gyeonggi-Do, Republic of Korea) and CGDerm (CGBio, Seongnam-Si, Gyeonggi-Do, Republic of Korea), and their size and thickness were determined according to the determined implant size, with sizes of 16 × 16, 18 × 18, and 20 × 20 cm and thickness ranging from 1.5 to 2.3 mm. A modified P-1 technique was applied by partially dissecting the superior portion of the pectoralis major using a Harmonic scalpel (Ethicon Inc, Cincinnati, OH, USA) to provide partial coverage of the implant's superomedial aspect. 11 All ADM-wrapped breast implants were placed in the prepectoral plane. In patients undergoing direct to implant surgery, two hemovac drains were placed: one directed toward the axilla and another toward the inframammary fold. In patients undergoing secondary bag change surgery, a single hemovac drain was placed toward the inframammary fold. Hemovac drains were anchored using 3–0 nylon sutures, subcutaneous closure was performed with 4–0 vicryl sutures, and skin closure was completed with 6–0 nylon sutures.

## Results

Among the five patients, four underwent immediate reconstruction, while one underwent a secondary bag change procedure. Except for one patient who was on oral medication for hypothyroidism, none had a history of diabetes mellitus or other significant medical conditions. None of the patients received preoperative chemotherapy, whereas one patient had undergone preoperative radiotherapy. This patient had previously received radiotherapy following partial mastectomy but underwent additional mastectomy due to recurrence ( Table 1 ). Regarding the type of mastectomy, two patients underwent robot-assisted nipple-sparing mastectomy, two underwent nipple-sparing mastectomy, and one underwent skin-sparing mastectomy with nipple–areolar complex sacrifice. All patients underwent ipsilateral sentinel lymph node biopsy, with no metastasis findings in the pathology reports. Postoperatively, one patient developed a hematoma immediately after surgery and required hematoma evacuation under general anesthesia on postoperative day 1. Two patients experienced hemovac obstruction issues that persisted for nearly 24 hours ( Table 2 ). *Corynebacterium striatum* was identified in the hemovac cultures or intraoperative specimens of all patients. The antibiotic susceptibility results commonly showed susceptibility only to vancomycin and gentamicin, while resistance was reported for all other antibiotics. Therefore, it was determined to be an infection caused by the same strain ( Table 3 ).

**Table 1:** Demographic characteristics of the patients

Patient no.	Age (yr)	Height (cm)	Weight (kg)	BMI (kg/m ^2^ )	Chest circumference (cm)	Previous medical history	Preoperative chemo therapy	Preoperative radiation therapy
1	62	165	60.4	22.1	73.5	No underlying disease	–	–
2	67	165.5	63.6	23.22	79.5	Hypothyroidism	–	–
3	51	165.7	53	19.3	68.5	Previous partial mastectomy history	–	+
4	51	157.4	61	24.62	82	No underlying disease	–	–
5	56	159.2	55.3	21.82	74.5	No underlying disease	–	–

Abbreviation: BMI, body mass index.

**Table 2:** Information related to surgery performed on patients

Patient no.	Mastectomy type	Lymph node procedure	Specimen weight (g)	Reconstruction surgery	Pocket plane	Implant profile	Type of ADM used	Complication
1	NSM	SLNB	374	DTI	Prepectoral	Moderate classic 255 cc	MegaDerm	Hematoma and mastectomy skin flap necrosis
2	RANSM	SLNB	Not measured	DTI	Prepectoral	Moderate classic 285 cc	MegaDerm	–
3	RANSM	SLNB	211	DTI	Prepectoral	Moderate classic 235 cc	MegaDerm	Hemovac obstruction
4	SSM	SLNB	257	Bag change	Prepectoral	High 425 cc	CGDerm	Hemovac obstruction
5	SSM	SLNB	270	DTI	Prepectoral	Moderate classic 190 cc	MegaDerm	Wound dehiscence

Abbreviations: ADM, acellular dermal matrix; DTI, direct-to implant; NSM, nipple-sparing mastectomy; RANSM, robot-assisted nipple-sparing mastectomy; SLNB, sentinel lymph node biopsy; SSM, skin-sparing mastectomy.

**Table 3:** Features and management of the patients with infections

Patient no.	Onset of signs of infection (POD)	Antibiotic use duration (days)	Date of surgical intervention (POD)	Date of implant exchange (POD)	Date of microorganism identification (POD)	Date of negative culture (POD)	Microorganism identified	Empirical antibiotics initially used	Antibiotics reported to be susceptible	The antibiotic switched to	Last follow-up date (POD)	Capsular contracture (Baker grade)
1	13	47	17, 58	17, 58	16	58	*C. striatum*	Cefazolin	Vancomycin, gentamicin	Vancomycin	378	I
2	22	25	22	–	26	–	*C. striatum*	Cefazolin	Vancomycin, gentamicin	Vancomycin	383	I
3	8	18	10, 17	17	10	20	*C. striatum*	Vancomycin	Vancomycin, gentamicin	Tigecycline	368	III
4	2	33	4, 14, 30	4	4, 33	12, 42	*C. striatum*	Vancomycin	Vancomycin, gentamicin		365	II
5	20	14	20	20	14, 23	30	*C. striatum*	Vancomycin	Vancomycin, gentamicin	–	305	I

Abbreviation: POD, postoperative day.

### Case 1 (Patient No. 1)

A 62-year-old woman underwent nipple-sparing mastectomy for left breast cancer followed by immediate breast reconstruction. As there was an additional lesion on the medial side of the inframammary fold, an additional skin excision of 4 × 3 cm was performed in that area. The mastectomy specimen weighed 374 g, and the implant used was moderate classic profile 255 cc.

Postoperatively, the patient developed breast bulging and a significant decrease in hemoglobin leading to bleeder ligation and hematoma evacuation on postoperative day 1. The patient showed good progress until postoperative day 12, but on day 13, erythema, localized heating sensation, tenderness at the skin excision site at medial side of the inframammary fold, and turbid discharge were observed. There was no fever, and the white blood cell count was within normal range (6,630 cells/μL), but the C-reactive protein (CRP) level was elevated (18.1 mg/L) along with an elevated erythrocyte sedimentation rate (ESR) of 35 mm/hr.

As soon as signs of infection were observed, we started administering IV cefazolin as empirical antibiotic therapy. After 3 days, a culture test identified *Corynebacterium striatum* , and based on the antibiotic susceptibility results, vancomycin was initiated after consultation with the infectious disease department.

Due to the development of mastectomy skin flap necrosis, surgery was planned. After removing the necrotic tissue, it was found that the ADM had been locally destroyed around the excision site. The infection in the pocket looked healthy, and after massive irrigation with betadine and antibiotics solution, the previously used ADM was wrapped around a new implant of the same size as the previous one, completing the surgery.

The culture test performed on the intraoperatively obtained tissue again identified the same strain of *Corynebacterium striatum* , and IV vancomycin treatment was continued. After 2 days of the surgery, the CRP decreased to 4.9 mg/L, and by 1 week, it had dropped to 0.8 mg/L, with noticeable improvement in heating sensation, tenderness, and other clinical and laboratory results, except for the discoloration of the mastectomy skin flap. The patient was discharged on day 26. Later, hemovac culture tests performed in the outpatient department showed no growth, and the drain was removed.

However, after 2 weeks, redness, skin thinning, and contracture reappeared. As a result, the patient was readmitted, but despite 2 weeks of IV vancomycin re-treatment, there was no improvement in clinical symptoms, and exploration surgery was performed on day 58. Localized fluid collection was drained at the area of erythema on the incision site. The breast pocket around the affected area was treated with betadine dry-up and underwent massive irrigation with betadine and antibiotic solution. The rest of the breast pocket was clean. A new implant was wrapped in a new ADM and replaced. After an additional week of vancomycin therapy, the clinical condition of the patient rapidly improved, and after confirming negative culture, the patient was discharged.

**Fig. 1:**
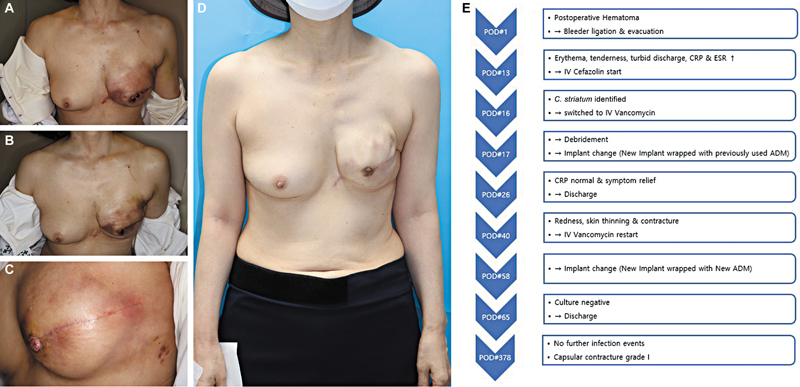
Photograph of the patient corresponding to Case 1. ( **A** ) Photograph showing the initial signs of infection. ( **B** ) Photograph showing the improved condition after the first implant change. ( **C** ) Photograph showing clinical symptoms suggestive of reinfection, accompanied by skin thinning. ( **D** ) Photograph showing the final resolution of the infection. ( **E** ) Flowchart summarizing the treatment course for Case 1.

Since discharge, there have been no further infection events, and on postoperative day 378, the discoloration of the mastectomy flap has minimalized, with the patient showing good results ( Fig. 1 ).

### Case 2 (Patient No. 2)

A 67-year-old woman underwent robot-assisted nipple-sparing mastectomy for right breast cancer followed by immediate breast reconstruction. The mastectomy specimen weight was not measured and the implant used was moderate classic profile 285 cc.

The patient had an uneventful discharge during the first hospitalization, with only mild color changes observed in the breast inferior pole, resembling congestion. At that time, all laboratory tests performed to rule out infection were normal, and the hemovac culture was also negative. Subsequently, during OPD follow-up, the hemovac drainage naturally reduced in volume with serous-colored fluid, leading to its removal.

However, redness persisted until postoperative day 22, and the condition worsened with the addition of heating sensation and swelling, raising suspicion of infection. The patient was readmitted and empirically treated with cefazolin, and a hemovac was reinserted. However, no fluid collection drainage was observed through the hemovac, and the drainage volume was minimal, leading to its removal after 3 days. Laboratory tests performed after admission showed CRP 4.9 mg/L, ESR 49 mm/hr, WBC 6,500 cells/μL, and no fever. Therefore, we decided to start antihistamine treatment, considering red breast syndrome, until the culture results were available.

However, the hemovac tip culture revealed *C. striatum* , and infection was diagnosed. IV vancomycin treatment was initiated. The clinical symptoms gradually improved, but on the second week, a fever occurred, accompanied by a decrease in WBC to 2,820 cells/μL, which had not been observed previously. This was diagnosed as drug fever in consultation with the infectious disease department, leading to the discontinuation of vancomycin, and the patient was discharged.

**Fig. 2:**
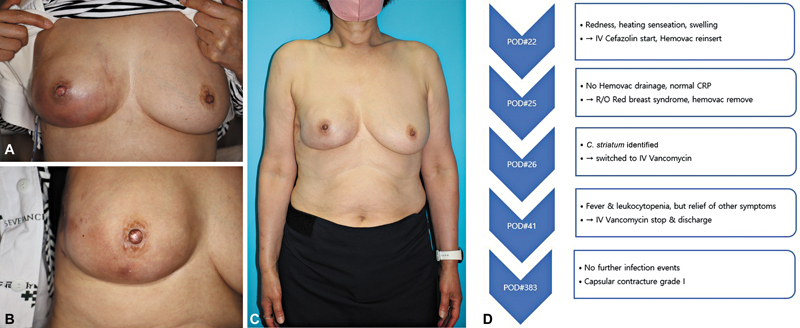
Photograph of the patient corresponding to Case 2. ( **A** ) Photograph showing the initial signs of infection. ( **B** ) Photograph showing the improved condition after 2 weeks of IV vancomycin treatment. ( **C** ) Photograph showing the final resolution of the infection. ( **D** ) Flowchart summarizing the treatment course for Case 2.

Since discharge, there have been no further infection events, and on postoperative day 383, the discoloration of the mastectomy flap minimalized, with the patient showing good results ( Fig. 2 ).

### Case 3 (Patient No. 3)

A 51-year-old woman underwent robot-assisted nipple-sparing mastectomy for left breast cancer followed by immediate breast reconstruction. The patient had previously undergone a partial mastectomy for breast cancer, with an incisional scar on the superolateral aspect. After the partial mastectomy, radiation therapy was performed; however, the cancer recurred, leading to the need for a subsequent mastectomy in this case. The mastectomy specimen weighed 374 g, and the implant used was moderate classic profile 211 cc.

The patient had no significant issues until postoperative day 2; however, on postoperative day 3, oozing was observed at the hemovac insertion site, and it was found that a hemovac obstruction had occurred. The obstruction was resolved through breast massage and hemovac line squeezing, and the patient was discharged without further issues.

From postoperative day 8, the patient developed fever, chills, tenderness, and redness at the previous partial mastectomy incision site, along with pain in the ipsilateral upper arm. Laboratory results showed CRP 196.8 mg/L, ESR 73 mm/hr, and WBC 22,450 cells/μL, prompting readmission and re-insertion of the hemovac. Empirical antibiotic treatment with IV vancomycin was initiated. A culture revealed *C. striatum* . Despite IV vancomycin treatment, the fever persisted, and there was no significant improvement in skin redness, with signs of impending necrosis. Due to ongoing active inflammation, surgical exploration was performed again after 7 days.

During exploration, focal ADM destruction and a small amount of clear fluid collection were observed, but the operative findings did not appear severe compared with the patient's symptoms. A debridement of the breast pocket was performed using a curette, followed by massive irrigation with betadine and antibiotics. A slightly smaller-sized implant was placed with a new ADM wrapped around it.

The patient initially planned to maintain IV vancomycin treatment for 2 weeks; however, a myalgia developed as a side effect. Consequently, in consultation with the infectious disease department, the antibiotic was switched to IV tigecycline. However, the patient experienced side effects such as nausea, diarrhea, and vomiting. Meanwhile, laboratory results returned to normal within 2 days, and the clinical symptoms showed rapid improvement, allowing the discontinuation of IV antibiotics and discharge from the hospital.

**Fig. 3:**
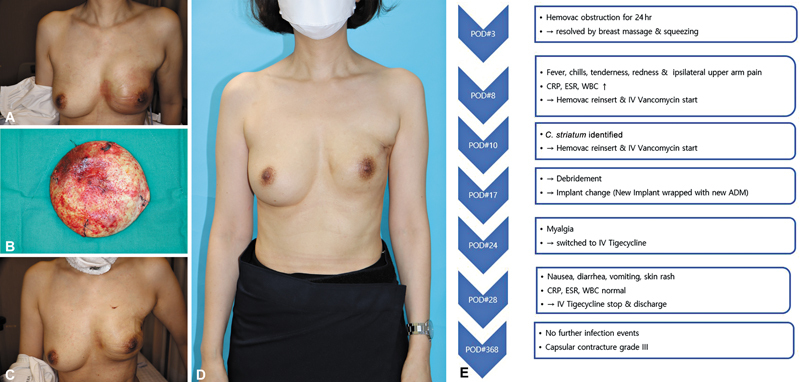
Photograph of the patient corresponding to Case 3. ( **A** ) Photograph showing the initial signs of infection. ( **B** ) Photograph of the ADM, which showed no significant destruction observed during surgical exploration. ( **C** ) Photograph showing the improved condition after the implant change. ( **D** ) Photograph showing the final resolution of the infection. ( **E** ) Flowchart summarizing the treatment course for Case 3.

Since discharge, there have been no further infection events, and on postoperative day 368, the patient has remained free of infection. Due to the history of previous radiation therapy and the current infection history, the patient has developed capsular contracture, and surgery for this condition is planned in the future ( Fig. 3 ).

### Case 4 (Patient No. 4)

A 51-year-old woman underwent a two-stage breast reconstruction using a tissue expander. Earlier, 4 months ago, she underwent a skin-sparing mastectomy for right breast cancer, followed by tissue expander insertion. The mastectomy specimen weighed 257 g, and since the patient desired contralateral breast augmentation, a 550-cc Mentor CPX ^TM^ 4 breast tissue expander Style 9300-Tall height (Mentor Worldwide LLC, Santa Barbara, CA, USA) was inserted. Prior to the secondary bag change, the tissue expander was inflated to 455 cc. During the bag change, a high-profile 425-cc implant was used, and contralateral breast augmentation was performed simultaneously.

Immediately after surgery, a hemovac obstruction issue occurred, followed by the onset of fever. Despite breast massage and hemovac line squeezing, the hemovac obstruction was resolved only on postoperative day 2. Afterward, redness, tenderness, and swelling were observed, and CRP was reported as 212.2 mg/L. Turbid discharge was drained from the hemovac, and a hemovac culture revealed *C. striatum* . Empirical IV vancomycin treatment was started, and on postoperative day 4, a rapid salvage procedure was performed. The ADM and breast pocket were dry-upped with betadine, and massive irrigation with betadine and antibiotic solution was performed, followed by implant change with a new one. Fever and laboratory values seemed to improve after about 3 days, but dehiscence of the incision site and turbid discharge persisted until postoperative day 14.

Subsequently, massive irrigation with betadine and antibiotics solution was performed, and wound revision was done. The operative findings showed focal destruction of the ADM around the incision, but no significant fluid collection or other major issues were noted. Hemovac culture was negative, and IV vancomycin treatment was continued for 2 weeks, leading to discharge.

However, on postoperative day 30, dehiscence and turbid discharge recurred. CRP was normal, and there was no fever. Due to persistent inflammation, the pocket became tight, and the entire breast pocket was dry-upped with betadine and subjected to massive irrigation with betadine and antibiotics solution. The implant was removed, and a tissue expander was inserted. IV vancomycin treatment was continued for 2 more weeks, and after confirming a negative hemovac culture and no further events, the patient was discharged.

**Fig. 4:**
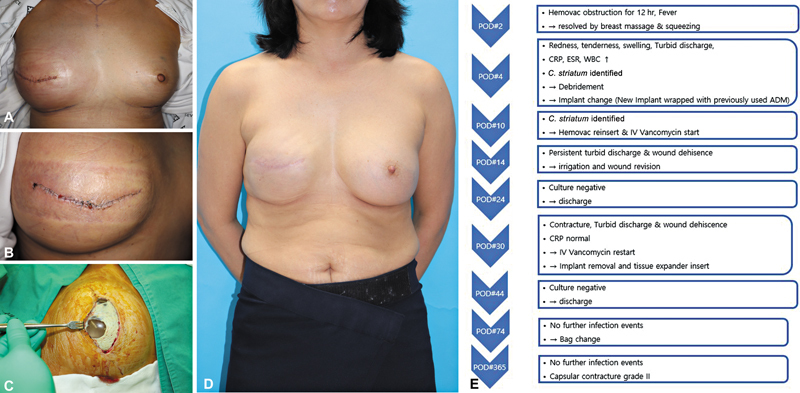
Photograph of the patient corresponding to Case 4. ( **A** ) Photograph showing the initial signs of infection. ( **B** ) Photograph showing dehiscence and turbid discharge despite early implant change. ( **C** ) Photograph of the ADM, which showed focal destruction, observed during surgical exploration. ( **D** ) Photograph showing the resolution of infection and the final completion of the bag change. ( **E** ) Flowchart summarizing the treatment course for Case 4.

A month after discharge, a bag change was performed, but pocket contracture developed, resulting in a smaller implant being inserted than originally planned, leading to a suboptimal result. Since then, there have been no further infection events, and on postoperative day 365, the patient has remained free of infection ( Fig. 4 ).

### Case 5 (Patient No. 5)

A 56-year-old woman underwent a skin-sparing mastectomy with nipple–areolar complex sacrifice for left breast cancer, followed by immediate breast reconstruction. The mastectomy specimen weighed 374 g, and the implant used was a moderate classic profile 255 cc. The mastectomy skin flap was closed using the purse-string suture method.

The patient was discharged without any notable issues after surgery. However, on postoperative day 10, delayed wound healing led to dehiscence, necessitating wound revision. At that time, there were no intraoperative findings suggestive of infection.

Despite this, the culture obtained during wound revision later revealed *C. striatum* . On postoperative day 20, redness appeared, and turbid discharge was observed at the dehiscence site. Empirical IV vancomycin treatment was promptly initiated. Additionally, for early salvage, surgical exploration was performed, involving betadine dry-up of the entire breast pocket and massive irrigation with betadine and antibiotic solution. The implant and ADM were replaced with new ones. During this procedure, no significant fluid collection was observed, and ADM destruction was minimal. During the implant change surgery, a wound culture was performed from within the breast pocket, a sterile site, and *Corynebacterium* was identified. Based on this finding, the case was diagnosed as a deep peri-prosthetic infection rather than a superficial surgical site infection. The patient completed a 2-week course of IV vancomycin, with subsequent culture confirming negative results, and was discharged.

**Fig. 5:**
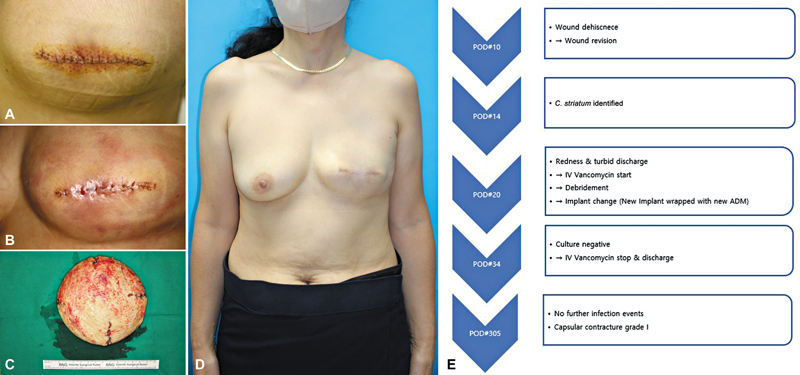
Photograph of the patient corresponding to Case 5. ( **A** ) Photograph showing the initial dehiscence. ( **B** ) Photograph showing the signs of infection including dehiscence and turbid discharge. ( **C** ) Photograph of the ADM, which showed no significant destruction, observed during surgical exploration. ( **D** ) Photograph showing the final resolution of the infection. ( **E** ) Flowchart summarizing the treatment course for Case 5.

Since discharge, there have been no further infection events, and on postoperative day 305, the patient is showing good results ( Fig. 5 ).

## Discussion

When *Corynebacterium* is identified in culture results, it is often considered part of the normal flora and assumed to be non-pathogenic, leading to its dismissal as contamination, which can potentially delay appropriate treatment. 12 In recent years, *C. striatum* has been increasingly identified in clinical cultures, demonstrating multidrug resistance and an expanding role in healthcare-associated infections, particularly in immunosuppressed patients. 13 14 15 16 Microbiological in vitro studies have shown that the ability to form a biofilm on different abiotic surfaces stands out as the most significant virulence factor possessed by this bacterium. 17 *C. striatum* forms colonies equally efficiently on both hydrophobic and hydrophilic surfaces, including polystyrene ureters. It could be expected that the *C. striatum* potential for biofilm formation is even higher in vivo due to the stimulatory effect of fibrinogen. 10 As a result, it has been frequently reported in medical device–related infections, including prosthetic joints, artificial valves, and catheters, and has been recognized as a challenging pathogen to manage. 5 6 7 However, although there are studies that briefly mention *Corynebacterium* as an uncommon pathogen accounting for only one or two cases among a larger number of infections, there is no previous literature specifically addressing the prevalence, characteristics, or treatment of *Corynebacterium* infections in breast implants. The fact that there are reports of catheter-related infections caused by *Corynebacterium* also suggests the need for increased attention to infections caused by this bacterium in breast reconstruction surgeries, particularly those involving hemovac drainage. 17 Furthermore, while there are many studies on infections related to implant-based breast reconstruction, most have primarily focused on common pathogens such as *Staphylococcus* and *Pseudomonas* . This report highlights that, although *Corynebacterium* infections are uncommon, they represent a clinically significant concern in breast implant–related infections. The consecutive occurrence of multiple cases within a short timeframe allowed for a more comprehensive observation of the clinical course and consistent application of salvage strategies. This experience enabled a more detailed description of effective management approaches, which may contribute to improving awareness and guiding clinical decision-making for this emerging pathogen.

Based on our experience, *Corynebacterium* infections are more likely to occur in situations where there is poor mastectomy flap perfusion, such as in patients with a history of preoperative radiation therapy, or when conditions favorable for bacterial growth, such as fluid congestion or seroma, are present. The initial symptoms that could raise suspicion of *Corynebacterium* infection commonly included erythema, swelling, and heating sensation, which can be easily mistaken for other infections. Other symptoms such as wound dehiscence, turbid drainage through the incision site or hemovac drainage, and CRP elevation were also observed, which were not significantly different from the typical symptoms of other infections. In the early stages, the symptoms tended to be localized rather than systemic, which aligns with observations made in other studies. 16 Notably, there were cases where the CRP and ESR levels did not show explosive increases, and the patients only had mild heating sensation, mild swelling, and erythema without other symptoms. The erythema also resembled the early congestion appearance seen in cases with poor flap circulation, making it difficult to differentiate from red breast syndrome and diagnose as an infection initially. However, over time, the condition responded to antibiotics, showing only slight discoloration and gradually improving, leading to a favorable outcome. Commonly, when the infection is not controlled and progresses, the skin becomes thinner, and patchy-like discoloration and erythema become more prominent. Dehiscence occurred at the suture site, or there was impending implant exposure. Fever or a significant increase in CRP were observed, and hemovac drainage continued in large amounts. In such cases, empirical antibiotic therapy was started, and as soon as possible, a hemovac drain was placed in the area suspected of fluid collection or in a dependent position for inpatient treatment.

There is no established consensus on empirical antibiotics for *Corynebacterium* infections, but current studies suggest options such as vancomycin, linezolid, gentamicin, and tigecycline. 18 19 20 As *Corynebacterium* is known to be a multidrug-resistant organism, all five of our patients shared a common antibiotic susceptibility to vancomycin and gentamicin, while they exhibited resistance to most other antibiotics. As a result, vancomycin was used for all patients, and in cases where the side effects of vancomycin necessitated a change in therapy, tigecycline was chosen based on the patient's antibiotic susceptibility results and in consultation with the infectious disease department. It is essential to thoroughly explain the potential for antibiotic-related side effects to patients, since intravenous antibiotics such as vancomycin and tigecycline can lead to adverse reactions including drug fever, myalgia, and other systemic symptoms. Although the clinical presentation did not suggest a destructive or severe infection, this infection did not respond rapidly to antibiotics, and clinical improvement took several days. After several days, there was a clear improvement in the clinical condition, with a reduction in hemovac drainage and no systemic symptoms. The patients were then treated with 2 weeks of IV antibiotics, and after confirming negative culture results, they were discharged and switched to oral antibiotics for further treatment. IV antibiotics were administered for an average duration of approximately 4 weeks to achieve infection control. Although this duration is relatively shorter compared with previous studies on medical device–related *Corynebacterium striatum* infections in other anatomical regions—which have reported a mean treatment duration of approximately 8 weeks 7 —favorable outcomes were achieved. This may be attributed to the fact that antibiotic therapy was not used in isolation but rather in combination with aggressive surgical management, including thorough debridement, copious irrigation, implant exchange, and replacement of the ADM. Considering that vancomycin, the primary antibiotic used, is associated with well-known adverse effects and can impose a significant burden on patients, the shorter treatment duration observed in our cases may offer meaningful clinical guidance. Notably, the shortest duration in our series was 2 weeks. Erythema and wound conditions generally improved rapidly following surgical intervention, although low-grade fever occasionally persisted for 1 to 2 days. Laboratory parameters normalized within 1 week, and follow-up cultures became negative on two consecutive tests.

If there is no significant improvement in symptoms despite antibiotic treatment, surgical intervention should be considered. In such cases, simply performing bactericidal irrigation or replacing the implant without changing the ADM may not lead to complete resolution. Studies on medical device–related *C. striatum* infections in other areas have shown that 40% of cases required implant change, and only 21% could be treated with isolated irrigation and debridement without implant change. 7 A two-stage salvage with implant removal followed by delayed re-reconstruction was not always considered necessary, and salvage was possible with a one-stage implant change in cases of severe inflammation. When surgery is required, it seems necessary to replace both the ADM and the implant with new ones while maintaining sensitive antibiotic therapy. In addition, thorough debridement of the breast pocket using a curette was performed in all implant-change surgeries, which is also considered a fundamental step in the management of infection. Surgical treatment involves significant costs, anesthesia, and patient discomfort, but it may not always lead to successful resolution, as seen in the cases presented. Therefore, obtaining the patient's informed consent is essential.

Capsular contracture is a well-known complication of implant-based breast reconstruction, and infection is one of its potential causes. Even if the infection is successfully treated, the clinical significance of this success would be diminished if capsular contracture were to develop after the resolution of infection. Although capsular contracture (grade III) developed in one case, this complication is presumed to be associated with the patient's prior history of partial mastectomy followed by radiotherapy. In most of the cases presented in this study, early detection and appropriate management of infection, the thorough debridement, massive irrigation with betadine and antibiotic solutions, and replacement of both the ADM and implant may have contributed to the absence of clinically significant contracture, leading to acceptable outcomes. Therefore, we believe that the treatment protocol described in this study represents a clinically viable option.

In this report, the same strain was repeatedly identified within a short period, and the antibiotic susceptibility testing showed similar patterns, suggesting that a single strain was responsible. However, since *Corynebacterium* is part of the normal skin flora, it was difficult to definitively determine the origin through cultures from the medical staff or other areas such as the operating room or hospital wards. Although *Corynebacterium* infections are typically known to occur in immunocompromised patients or as opportunistic infections, 9 10 all our cases involved patients with no significant medical history other than breast cancer. This finding highlights the importance of not dismissing *Corynebacterium striatum* as mere contamination in patients without a particular history (or immunosuppression), but rather considering the possibility of infection and thoroughly investigating its role. To distinguish true infection from contamination, a comprehensive evaluation is necessary, including clinical correlation, identification of the anatomical site of infection, and, when possible, repeated isolation from sterile sources. To minimize such infections, it is critical to adhere to aseptic principles in the operating room and ensure that sterile gloves are worn during dressing changes, keeping the dressing procedure clean. 21

*Corynebacterium striatum* infections are often dismissed as contamination, but they can present significant clinical challenges, particularly in implant-based breast reconstruction. Based on our experience, the initial symptoms include localized erythema, swelling, and mild warmth, which can be easily confused with early signs of congestion or red breast syndrome, making early diagnosis difficult. As the infection progresses, more severe symptoms such as wound dehiscence, turbid drainage, and elevated CRP levels can develop. Diagnosis was confirmed through cultures from hemovac drainage or wound discharge, and this bacterium often demonstrates multidrug resistance. Empiric antibiotic therapy typically begins with vancomycin, and alternative agents like tigecycline may be used if side effects occur. In more severe or persistent cases, surgical intervention, including massive irrigation with a betadine and antibiotic solution, thorough debridement of the breast pocket using a curette, and implant replacement along with ADM change, may be necessary. Once the antibiotic susceptibility results from the cultures are reported, a change in antibiotics may be necessary. Generally, treatment involves at least 2 weeks of IV antibiotics, followed by a switch to oral antibiotics once the culture results are confirmed to be negative.
